# The chromosomal instability 25 gene signature is identified in clear cell renal cell carcinoma and serves as a predictor for survival and Sunitinib response

**DOI:** 10.3389/fonc.2023.1133902

**Published:** 2023-05-01

**Authors:** Chang Wang, Xin Qin, Wei Guo, Jing Wang, Li Liu, Zhiqing Fang, Huiyang Yuan, Yidong Fan, Dawei Xu

**Affiliations:** ^1^ Department of Emergency, The Second Hospital of Shandong University, Jinan, China; ^2^ Department of Emergency, Qilu Hospital of Shandong University, Jinan, China; ^3^ Department of Urologic Oncology, Division of Life Sciences and Medicine, University of Science and Technology of China, The First Affiliated Hospital of University of Science and Technology of China (USTC), Hefei, China; ^4^ School of Nursing, Beijing University of Chinese Medicine, Beijing, China; ^5^ Department of Medicine, Division of Hematology, Bioclinicum and Center for Molecular Medicine, Karolinska Institute and Karolinska University Hospital Solna, Stockholm, Sweden

**Keywords:** ccRCC, chromosomal instability, CIN25, prognosis, Sunitinib, telomere

## Abstract

**Background:**

Chromosomal instability (CIN) is a cancer hallmark and it is difficult to directly measure its phenotype, while a CIN25 gene signature was established to do so in several cancer types. However, it is currently unclear whether there exists this signature in clear cell renal cell carcinoma (ccRCC), and if so, which biological and clinical implications it has.

**Methods:**

Transcriptomic profiling was performed on 10 ccRCC tumors and matched renal non-tumorous tissues (NTs) for CIN25 signature analyses. TCGA and E-MBAT1980 ccRCC cohorts were analyzed for the presence of CIN25 signature, CIN25 score-based ccRCC classification, and association with molecular alterations and overall or progression-free survival (OS or PFS). IMmotion150 and 151 cohorts of ccRCC patients treated with Sunitinib were analyzed for the CIN25 impact on Sunitinib response and survival.

**Results:**

The transcriptomic analysis of 10 patient samples showed robustly upregulated expression of the CIN25 signature genes in ccRCC tumors, which were further confirmed in TCGA and E-MBAT1980 ccRCC cohorts. Based on their expression heterogeneity, ccRCC tumors were categorized into CIN25-C1 (low) and C2 (high) subtypes. The CIN25-C2 subtype was associated with significantly shorter patient OS and PFS, and characterized by increased telomerase activity, proliferation, stemness and EMT. The CIN25 signature reflects not only a CIN phenotype, but also levels of the whole genomic instability including mutation burden, microsatellite instability and homologous recombination deficiency (HRD). Importantly, the CIN25 score was significantly associated with Sunitinib response and survival. In IMmotion151 cohort, patients in the CIN25-C1 group exhibited 2-fold higher remission rate than those in the CIN25-C2 group (*P* = 0.0004) and median PFS in these two groups was 11.2 and 5.6 months, respectively (*P* = 7.78E-08). Similar results were obtained from the IMmotion150 cohort analysis. Higher EZH2 expression and poor angiogenesis, well characterized factors leading to Sunitinib resistance, were enriched in the CIN25-C2 tumors.

**Conclusion:**

The CIN25 signature identified in ccRCC serves as a biomarker for CIN and other genome instability phenotypes and predicts patient outcomes and response to Sunitinib treatment. A PCR quantification is enough for the CIN25-based ccRCC classification, which holds great promises in clinical routine application.

## Introduction

Sporadic clear cell renal cell carcinoma (ccRCC) is the major subtype of renal cell carcinoma (RCC) (up to 80% of all RCCs) and originates from the epithelial cells in the nephron (1–3). Most patients are diagnosed early when tumors are localized, and thus successfully removed *via* nephrectomy, but the disease will eventually recur in about 30% of them post-surgery (2, 4). Clinical and pathological variables have been traditionally applied to stratify recurrence risk and survival, however, there exist certain limitations (4). To further improve the robustness of ccRCC prognostication, molecular biomarkers, such as multigene expression signature models, have recently been established to make molecular classifications or to combine with clinic-pathological factors for stratifications (5–11). Despite so, a substantial gap remains between all the models currently applied in the clinic and the prediction accuracy. Therefore, looking for more reliable prognostic factors is an unmet demand.

Metastasis readily occurs in approximately 1/3 of ccRCC patients at diagnosis, which requires adjuvant treatments (4, 12, 13). These same interventions are also requisite for patients with recurrent ccRCC or even patients with localized ccRCC (12, 14, 15). However, ccRCC tumors are intrinsically insensitive to conventional chemo- and radio-therapies (12, 14). Fortunately, over the last decades, targeted therapies, immunotherapies, and other multi-therapeutic modalities have been developed, which has revolutionized ccRCC treatment landscapes (14). For instance, immune checkpoint inhibitors (ICIs) are used to target immune checkpoint proteins PD-1/PDL-1 and/or CTLA4, thereby boosting anti-cancer immune response and showing a great efficacy in ccRCC (14, 16). Targeted therapeutic drugs, which mainly includes tyrosine kinase receptor inhibitors (TKRis), such as the small molecule Sunitinib, have been approved for the first-line treatment of metastatic ccRCC (13–15, 17). However, subsets of patients do not respond or develop resistance to ICI and/or TKRi treatments (6, 13–15, 17). Distinguishing responders from non-responders should be clinically important for personalized interventions of ccRCC.

**Figure 1:**
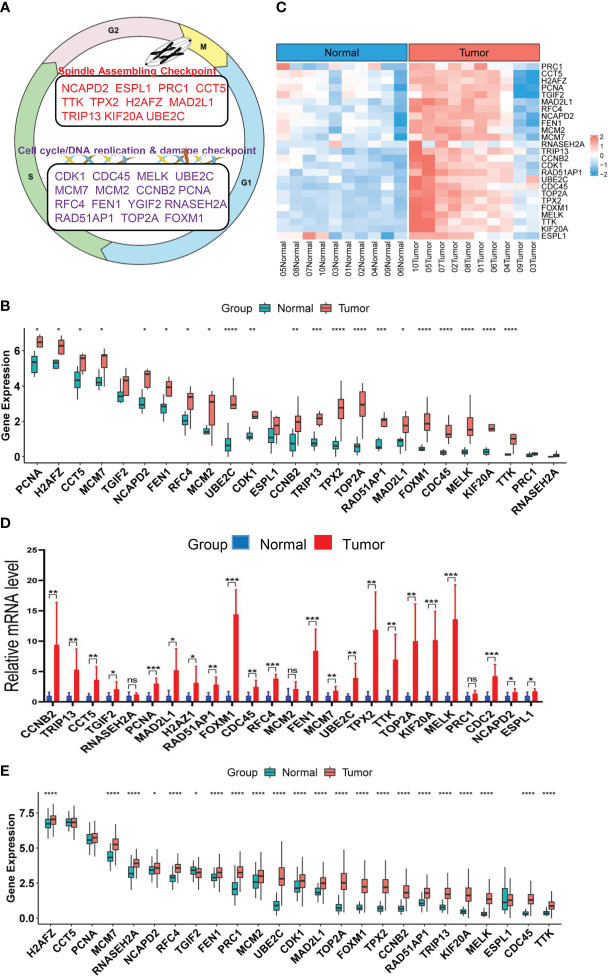
Upregulation of CIN25 genes in ccRCC tumors. **(A)** The CIN25 signature genes and their function. **(B)** Upregulated expression of 25 genes included in the CIN25 signature in primary ccRCC tumors. Tumors and matched non-tumorous tissues (NTs) from 10 patients were analyzed for transcriptomic profile and expression levels of CIN25 genes were expressed as Transcripts Per Million (TPM) counts. **(C)** The heatmap showing CIN25 ssGSEA scores between 10 ccRCC tumors and their matched NTs. **(D)** The qPCR validation of upregulated CIN25 gene expression in primary ccRCC tumors. Paired specimens from 9 ccRCC patients were analyzed for mRNA levels of CIN25 genes. mRNA levels of target genes were based on 2(−ΔΔCT) values and normalized by β-actin expression. **(E)** Differences in expression of 25 CIN25 signature genes between 530 ccRCC tumors and 72 NTs in the TCGA cohort. TPM was used for expression level. *, **, *** and **** indicate *P* values <0.05, 0.01, 0.001 and 0.0001, respectively. ns, Not significant.

It has long been documented that aneuploidies, or somatic copy number alterations (SCNAs), are associated with ccRCC outcomes, including recurrence, and metastasis, survival and drug resistance (4, 18–21). Therefore, aneuploidies and SCNAs have been used as genomic prognostic biomarkers in ccRCC (19–21). Mechanistically, aneuploidies or SCNAs are primarily driven by chromosomal instability (CIN), the cancer hallmark event resulting from persistent high-rates of chromosome mis-segregations during mitosis (22–25). The direct assessment of the CIN phenotype is difficult, and Carter et al. identified a 25 gene expression signature of CIN, so-called CIN25, for the CIN measurement (22). The genes included in the CIN25 are involved in spindle assembly checkpoint signaling, proliferation, and DNA replication and repair (**Figure 1A**) (22). By calculating their expression score, the authors showed a strong correlation between the CIN25 score and levels of CIN (22). The CIN25 was further observed to serve as a prognostic factor in breast, lung and several other cancers (22, 26). It is currently unclear whether this CIN25 signature is present in ccRCC, and if so, whether it has any clinical implications. Moreover, because CIN plays an important part in the cancer evolution, progression, and drug resistance (23), it is warranted to elucidate the relationship between CIN25 and targeted therapies of ccRCC. The present study is thus designed to address these issues. To this end, we performed the transcriptomic profiling in ccRCC tumors together with their matched renal tissues and analyzed TCGA and other cohorts of ccRCC.

## Materials and methods

### Primary ccRCC tumor specimens and their matched renal noncancerous tissues

Nineteen patients with ccRCC, diagnosed at Qilu Hospital of Shandong University, were randomly recruited and their clinical information was listed in **Table S1**. Tumors and their matched NT specimens were collected from these patients who underwent nephrectomy. All the samples were stored in nitrogen tanks until use. The study was approved by the Institutional review board of Qilu Hospital of Shandong University (#KYLL-2021(KS)-192) and the signed informed consent was obtained from all patients.

### RNA extraction and RNA sequencing

RNA was extracted from primary tissues and cells using a RNAfast2000 kit (Fastagen) and quality control was performed using NanoDrop ND-1000 (Thermo Fisher Scientific). RNA sequencing was performed on 10 paired specimens. Sequencing libraries were generated using NEBNext^R^ Ultra™ RNA Library Prep Kit (New England Biolabs) according to the manufacturer’s recommendation. RNA sequencing was carried out using Illumina HiSeq 4000 sequencer at Metware Biotechnology (Wuhan, China). Paired-end reads were quality controlled by Q30 and Cutadapt software (v 1.9.3) was used to remove low-quality reads and 3’ adaptor-trimming. Hisat2 (v 2.0.4) was further used to align clean reads from sequencing, and sequencing depth and gene length were adjusted by Fragments Per Kilobase of transcript per Million (TPM) fragments mapped. The sequencing data were deposited in the GEO database (GSE217386).

### Reverse transcription and qPCR analysis

The qPCR evaluation of CIN25 gene expression was performed on paired specimens from 9 patients with ccRCC. cDNA was synthesized using a PrimeScript™ RT reagent Kit (TAKARA). qPCR was carried out using SYBR Green of RT Master Mix (TAKARA) to assess mRNA levels of target genes based on 2(−ΔΔCT) values. β-actin mRNA levels were used as the internal control for normalization of target gene expression. All the primers were synthesized at Wuhan Genecreate Biotech (Wuhan, China) and primer sequences are listed in **Table S2**.

### Data collection and processing of ccRCC tumors

The TCGA cohort of ccRCCs included 530 tumor samples with survival information available and 72 renal NTs. Patient clinical data were summarized in **Table S3** (27). Transcriptome, mutation, copy number variations (CNAs) and clinical-pathological data were downloaded from https://gdc.cancer.gov/. One hundred and one patients with ccRCC were in the E-MTAB-1980 cohort (28), and RNA array and clinical information were downloaded from http://www.ebi.ac.uk. Patient clinical characteristics were listed in **Table S4**. For RNA sequencing data, mRNA abundances were expressed as TPM. For array results (determined by 4×44K v2 microarray kit) from the E-MTAB-1980 cohort, probe-set values were used to quantify mRNA levels. ccRCC patients receiving Sunitinib treatments were contained in IMmotion150 (**Table S5**) (29, 30) and IMmotion151 (**Table S6**) trials (31, 32). Expression differences in CIN25-containing 25 genes were compared between ccRCC tumors and NTs in the TCGA cohort. For RNA expression, log2(TPM+1) based on RNA sequencing data was from https://gdc.cancer.gov/ as stated above. Protein expression data was obtained from Clinical Proteomic Tumor Analysis Consortium (http://ualcan.path.uab.edu/index.html).

### CIN25 signature

The CIN25 gene signature includes the following genes responsible for spindle assembling/checkpoint, DNA damage checkpoint and cell cycle regulation: NCAPD2, ESPL1, CDK1, MELK, PRC1, KIF20A, TOP2A, TTK, TPX2, UBE2C, MCM7, MCM2, RFC4, FEN1, CDC45, FOXM1, RAD51AP1, H2AFZ, MAD2L1, PCNA, RNASEH2A, TGIF2, CCT5, TRIP13 and CCNB2 (22) (**Figure 1A**). The CIN25 score for each sample were expressed as mean Z-scores based on the Z-normalized mRNA level of 25 CIN-related genes above. We also calculated the CIN25 score based on single sample gene set enrichment analysis (ssGSEA) to confirm the accuracy of the Z-score method and other purposes.

### Copy number alterations and aneuploidy score analysis

Somatic CNAs were downloaded from https://xenabrowser.net/. CNA plots were made using R package ‘oncoPrint’ in ‘ComplexHeatmap’. Aneuploidy scores were the sum total of altered (amplified or deleted) chromosome arms. TMB is defined as the number of non-silent mutations per million bases and the data were downloaded from https://xenabrowser.net/.

### Analyses for proliferation, cancer stemness, Epithelial–mesenchymal transition, angiogenesis and telomerase score

Proliferation statuses were estimated using expression levels of Ki-67 mRNA and cell cycle scores, respectively. ccRCC cell cycle, stemness, EMT and angiogenesis signature scores were calculated based on ssGSEA or as the median z-score of genes included in each signature for each sample. These signatures are as follow: Angiogenesis: VEGFA, KDR, ESM1, PECAM1, ANGPTL4 and CD34 (33). Cell Cycle: CDK2, CDK4, CDK6, BUB1B, CCNE1, POLQ, AURKA, KI-67 and CCNB2 (34, 35). EMT: VIM, CDH2, FOXC2, SNAI1, SNAI2, TWIST1, FN1, ITGB6, MMP2, MMP3, MMP9, SOX10, GCS, CDH1, DSP and OCLN (36).

### Telomere length and telomerase activity assessments

Telomere length data in the TCGA cohort of ccRCCs were from Bartheal et al. (37). Telomerase activity was evaluated using the telomerase score based on expression levels of the following 10 telomerase factors: TERT, TERC, DKC1, NHP2, NOP10, TCAB1, GAR1, NVL, RUVBL1 and RUVBL2 (38).

### GSEA analysis

GSEA (http://www.gsea-msigdb.org/) analyses were performed to enrich KEGG pathways and hallmarks in two CIN25 subtypes of ccRCC tumors. *P <*0.05 and False discovery rate (FDR) <0.05 was considered statistically significant.

### Nomograms for survival prediction

Cox regression analysis was conducted to assess the effect of the CIN25 cluster and clinical variables on survival. Then according to multivariate Cox regression analysis results, we constructed predictive nomograms including CIN25 and stage to predict 1-, 3-, and 5-year OS and/or PFS). Predicted survival of the nomogram against observed ones was plotted using the calibration curve. All nomograms and assessments of their predicative powers were made using R package regplot. Time-dependent Receiver Operator Characteristic (ROC) curves were used to determine sensitivity and specificity of OS and PFS predictions. Time-dependent ROCs and AUCs were made using Rpackage timeROC.

### Statistical analysis

All statistical analyses were carried out using R package version 4.0.5. Wilcox and K-W sum tests were used for analysis of differences between two groups and among multi groups, respectively. Spearman’s Rank-Order Correlation coefficient was applied to determine correlation coefficients r between two variables. Survival analyses were made using log-rank test. The Survival and Survminer packages were employed to draw Kaplan–Meier survival curves for visualization of OS and PFS. Univariate and multivariate Cox regression analyses were used to determine the effect (HR and 95% CI) of various quantitative predictor variables on OS and PFS. *P* < 0.05 were considered as statistically significant.

## Results

### Robust upregulation of the CIN25 signature genes in primary ccRCC tumors

Although aneuploidies and SCNAs have been well documented in ccRCCs, it remains unclear whether there exists the CIN25 signature as identified in other tumor types. We thus probed this issue first. RNA sequencing was performed on primary ccRCC tumors and their matched NTs from 10 patients who underwent nephrectomy. Expression levels of 25 genes in the CIN25 signature were evaluated in both tumors and NTs. As shown in **Figure 1B**, tumors exhibited significantly upregulated expression of 21/25 genes. The analysis of CIN25 ssGSEA in these samples further unraveled enhanced CIN25 levels in tumors (**Figure 1C**). For validation, we did qPCR-based expression analyses of these 25 genes in paired tumors and NTs from 9 patients, and largely similar results were obtained (**Figure 1D**). To confirm this finding obtained from our small patient cohorts, we analyzed the TCGA ccRCC sequencing data for their CIN25 signature expression. The comparison between 530 tumors and 72 NTs revealed significantly higher mRNA levels of 22/25 genes in tumors than in NTs (**Figure 1E**). Protein information was available in 20 of 25 genes, and protein levels were similarly higher in tumors, which is consistent with the transcriptomic analysis data (**Figure S1**).

### CIN25 expression-based classification of ccRCCs

**Figure 2:**
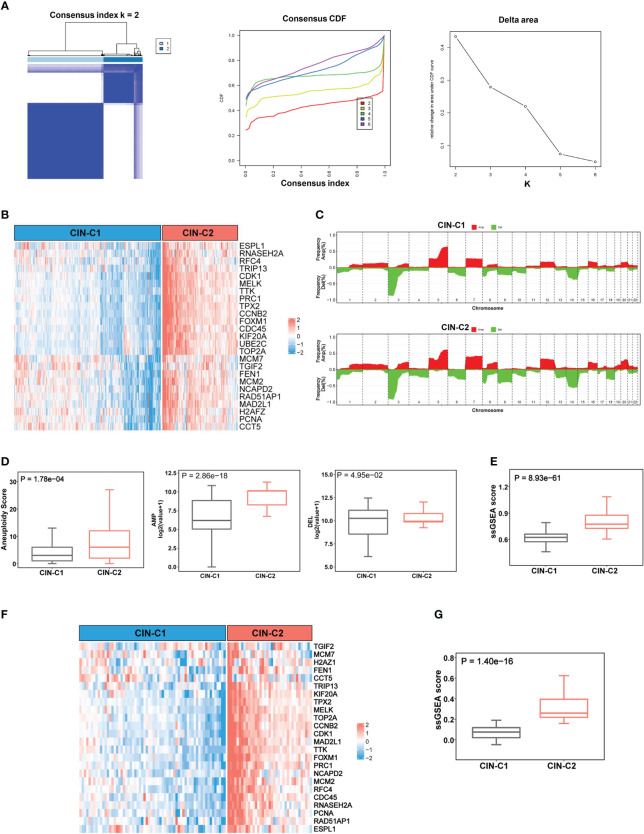
CIN25 signature-based classification of ccRCCs. **(A)** Consensus clustering of ccRCC tumors according to expression of CIN25 genes. A two-cluster classification of ccRCC tumors was optimal CIN25 signature-based clustering based on the K value from nonnegative matrix factorization. CDF: Cumulative distribution function. **(B)** TCGA ccRCC tumor clustering. Tumors were categorized into two clusters: CIN25-C1 (low) and CIN25-C2 (high). **(C)** Global copy number alterations (CNAs) in CIN25-C1 and CIN25-C2 tumors. The plots show frequencies of gain/amplification (Red) and deletion (Green) in 22 chromosomes. Top and bottom: CIN25-C1 and CIN25-C2, respectively. **(D)** Differences in the total aneuploidy score, and amplified and deleted scores between CIN25-C1 and CIN25-C2 tumors. **(E)** Differences in CIN25 ssGSEA score between CIN25-C1 and CIN25-C2 tumors. **(F)** CIN25 signature-based clustering of ccRCC tumors in the E-MTAB1980 cohort. **(G)** Differences in CIN25 ssGSEA score between CIN25-C1 and CIN25-C2 tumors in the E-MTAB1980 cohort.

The results above demonstrate highly upregulated expression of almost all CIN25 genes in ccRCC tumors, however, a significant heterogeneity was observed among them. To determine whether ccRCC tumors could be classified based on the CIN25 expression score, we performed consensus cluster analyses of the TCGA cohort. Nonnegative matrix factorization clustering of CIN25 mRNA data showed consistency K = 2, indicating that a two-cluster classification was optimal (**Figure 2A**). In a total of 530 tumors, CIN25-cluster 1 (CIN25-C1, low CIN level) and cluster 2 (CIN25-C2, high CIN level) were 350 (66%) and 180 (34%), respectively (**Figure 2B**). Because the CIN phenotype is characterized by the presence of aneuploidy, we further compared global CNAs, and calculated aneuploidy, amplified and deleted scores between two CIN25 clusters (**Figures 2C, D**). Indeed, the aneuploidy score was significantly higher in CIN25-C2 tumors (CIN25-C1 vs CIN25-C2, *P* = 1.78E-04) (**Figures 2C, D**). Interestingly, the amplified score was more robustly higher in the CIN25-C2 tumors than in CIN25-C1 ones (CIN25-C1 vs CIN25-C2, the amplified and deleted scores, *P* = 2.86E-18 and 4.95E-02, respectively) (**Figure 2D**). Moreover, we also calculated CIN25 ssGSEA score of each tumor based on the expression of 25 genes and observed a drastically higher CIN25 ssGSEA score in CIN25-C2 tumors (**Figure 2E**). To validate the CIN25 clustering classification of ccRCC tumors, we carried out the same analysis of the E-MTAB1980 ccRCC cohort, and tumors were readily categorized into two distinct CIN25 clusters, with higher CIN25 ssGSEA scores in CIN25-C2 tumors (**Figures 2F, G**).

### Association between CIN25 subtypes and clinic-pathological variables

**Figure 3:**
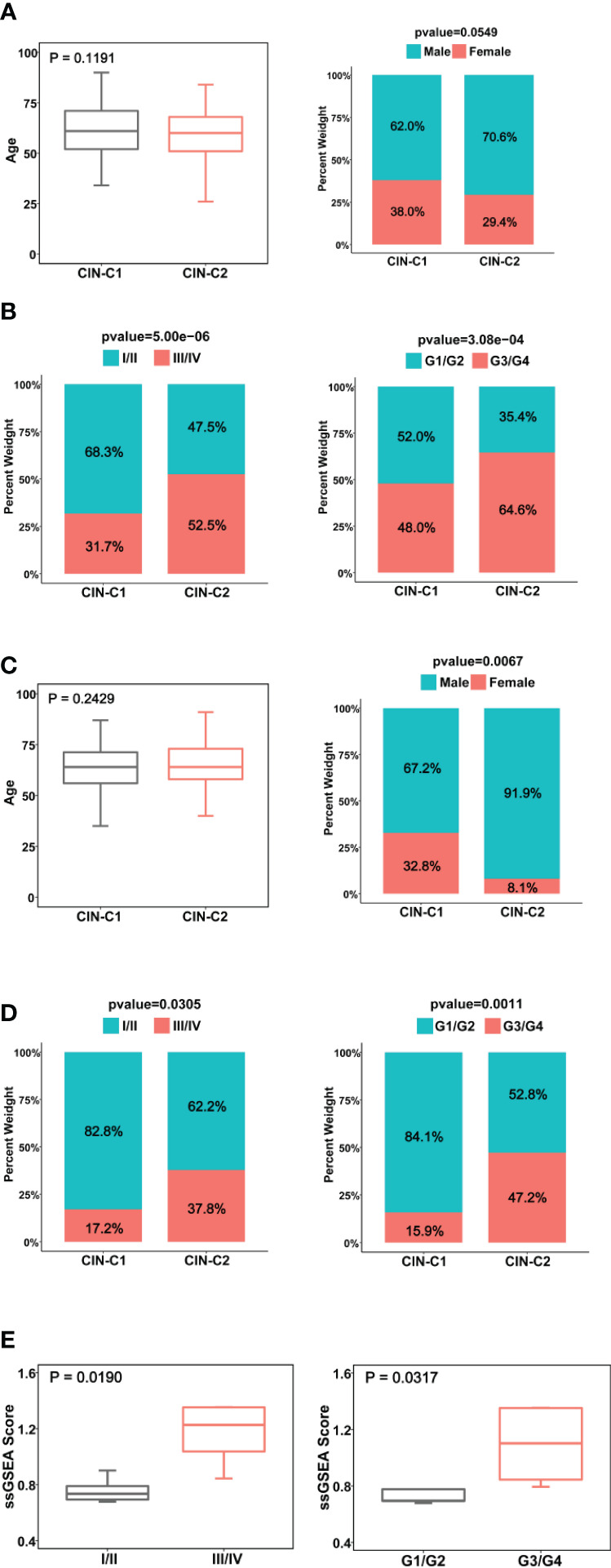
Association between CIN25 subtypes and clinic-pathological characteristics in ccRCCs. **(A, B)** The TCGA cohort. CIN25 subtypes were associated with stages and grades, but not age and gender. **(C, D)** The E-MTAB1980 cohort. CIN25 subtypes were associated with stages and grades but not age. More female patients were in the CIN25-C1 group. **(E)** The present cohort of 10 patients. Advanced stages and grades of ccRCC tumors exhibited significantly higher CIN25 ssGSEA scores. The CIN25 ssGSEA score was calculated as described in the Method.

We next determined the potential association between CIN25 subtypes and clinic-pathological variables in ccRCC tumors. We first examined the distribution of two clusters between two genders and different age groups (≥60 and <60 years) in the TCGA cohort and did not observe significant differences, although male patients had a slightly higher frequency of CIN25-C2 than did females (38.6% vs 29.4%, *P* = 0.055) (**Figure 3A**). CIN25-C2 was more frequently observed in higher-stage (*P* = 5E-06) and higher-grade tumors (*P* = 0.007) (**Figure 3B**). Very similar results were obtained from the analysis of the E-MTAB1980 cohort (**Figures 3C, D**).

We further performed the same analysis of 10 ccRCC patients whose tumors were with transcriptomic profiling. Because 10 tumors were too few to make a CIN classification, we calculated ssGSEA score to express CIN25 levels in each tumor. The CIN25 ssGSEA score was significantly increased in higher-stage (III/IV vs I/II, *P* = 0.019) and grade (III/IV vs I/II, *P* = 0.032) tumors (**Figure 3E**), which was consistent with the result obtained from the TCGA patient analysis.

### Telomere length, telomerase and genomic aberrations in CIN25 subtypes of ccRCC tumors

**Figure 4:**
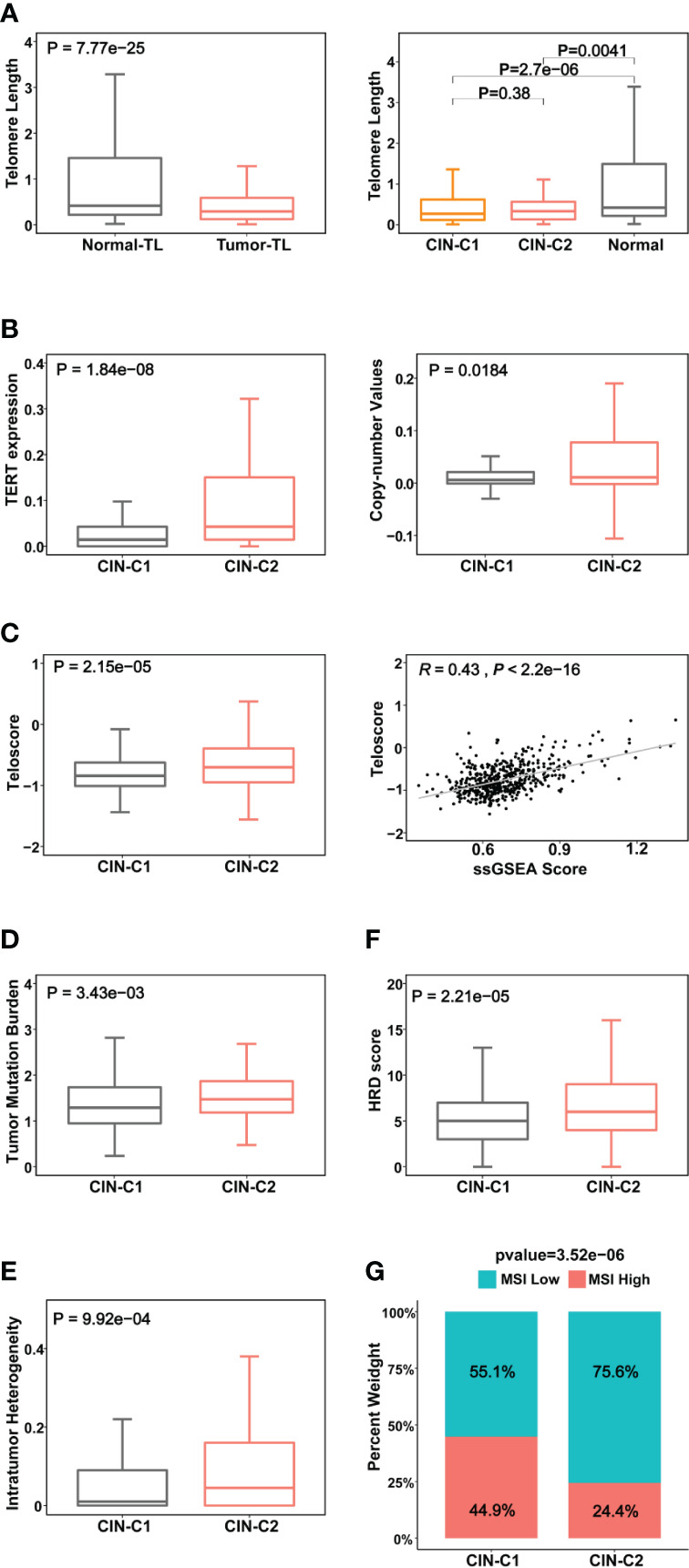
Association between CIN25 subtypes and telomere length, telomerase and other genomic alterations in ccRCCs. The TCGA cohort of ccRCCs were analyzed. Telomere length data were from reference 33. **(A)** Telomere shortening occurred in ccRCC tumors independently of CIN25 subtypes. Left panel: ccRCC tumors had significantly shorter telomeres than did renal nontumorous tissues (NTs). Right panel: Both CIN25-C1 and C2 tumors had similar telomere length, shorter than NTs. **(B)** Robustly higher TERT expression (left) and increased *TERT* copy numbers (right) in CIN25-C2 tumors. **(C)** Left panel: Significantly higher levels of telomerase activity, as assessed using the telomerase score in CIN25-C2 tumors. Right panel: The strong correlation between telomerase score and CIN25 ssGSEA score in ccRCC tumors. **(D–G)** CIN25-C2 tumors coupled with higher levels of other types of genomic instability. Higher tumor mutation burden (TMB) **(D)**, intratumoral heterogeneity (ITH) **(E)**, homologous recombination deficiency (HRD) **(F)** and microsatellite instability (MSI) **(G)** in CIN25-C2 tumors.

It is well established that telomere dysfunction drives CIN in oncogenesis (39). We thus sought to determine whether telomere length was altered in the TCGA ccRCC cohort. Telomeres were significantly shorter in tumors than in matched NTs (**Figure 4A**), but there was no statistically significant difference in telomere length between CIN25-C1 and C2 subtypes (**Figure 4A**). Because telomeric DNA is synthesized by telomerase, while telomerase activity is primarily governed by its catalytic component telomerase reverse transcriptase (TERT) (40), we further compared TERT expression and telomerase activity between CIN-C1 and C2 tumors. As shown in **Figure 4B**, TERT mRNA levels were significantly higher coupled with the increased frequency of TERT copy number gain in CIN25-C2 tumors (C2 vs C1 for TERT mRNA and copies: *P* = 1.84E-08, and 0.018, respectively). Telomerase activity, as determined using telomerase score (38), increased markedly in the CIN25-C2 tumors compared with that in CIN25-C1 tumors (*P* = 2.15E-05) (**Figure 4C**). Moreover, there was a significantly positive correlation between telomerase and CIN25 ssGSEA scores (*R* = 0.43, *P <*2.22E-16) (**Figure 4C**).

CIN is one subtype of genomic instability, whereas the later also includes several other forms of genomic aberrations such as nucleotide instability (NIN), microsatellite instability (MSI), homologous recombination deficiency (HRD), etc. (41). Thus, we further addressed the association of CIN25 clusters with the following important alterations: (i) Tumor mutation burden (TMB) (*P* = 0.034) (**Figure 4D**). Moreover, we compared the top 10 mutated genes between two subtypes. As expected, VHL, PBRM1, BAP1, MTOR and SETD2 are among the top mutated genes in both subtypes, however, significantly higher mutated frequencies of BAP1 and SETD2 were observed in the CIN25-C2 tumors (BAP1 and SETD2: *P* = 0.0003 and 0.018, respectively (**Figure S3**). In addition, KDM5C mutation was more frequent in the CIN25-C1 tumors (*P* = 0.04). (ii) Intratumor genetic heterogeneity (*P* = 0.01) (**Figure 4E**). (iii) HRD (*P* = 0.0002) (**Figure 4F**). (iv) MSI (*P* = 0.00004) (**Figure 4G**). CIN25-C2 tumors exhibited significantly higher levels or frequencies of all the aberrations analyzed above.

### CIN25 clusters for prediction of ccRCC patient survival

**Figure 5:**
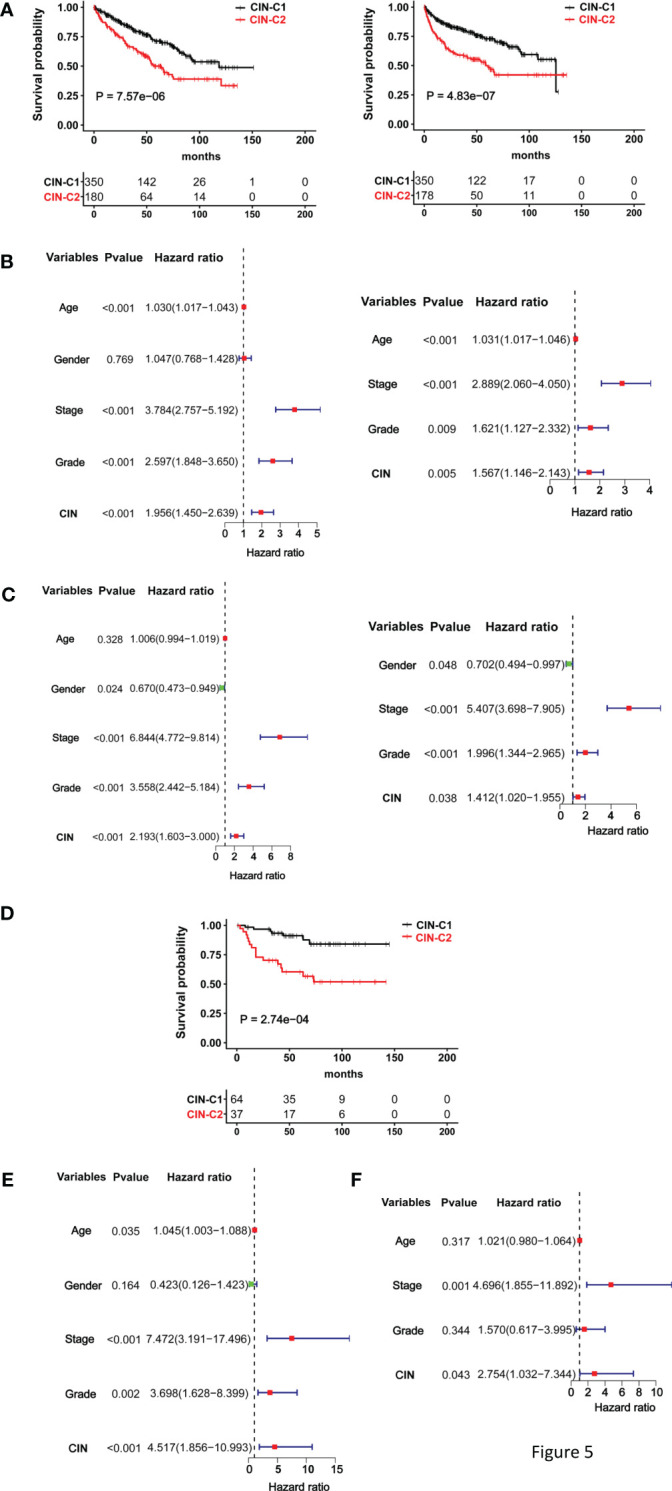
The CIN25 subtypes for survival prediction in ccRCCs. **(A–C)** The TCGA cohort analysis and **(D, E)** The E-MTAB1980 cohort analysis. **(A)** Significantly shorter overall and progression-free survival (OS and PFS) in the CIN25-C2 group. Left and right panel: OS and PFS, respectively. **(B)** Univariate and multivariate COX regression analyses of OS for the TCGA ccRCCs. **(C)** Univariate and multivariate COX regression analyses of PFS for the TCGA ccRCCs. **(D)** Significantly shorter OS in the CIN25-C2 group in the E-MTAB1980 cohort. **(E, F)** Univariate and multivariate COX regression analyses of OS for the E-MTAB1980 cohort.

We then wanted to assess whether this CIN25 classification system could predict patient survival. The TCGA cohort of 530 ccRCC patients was first evaluated as the discovery set. Log-rank test analysis unravelled that those patients in the CIN25-C2 group had significantly shorter OS and PFS, as shown by Kaplan–Meier survival curves (*P* = 7.57E-06 and 4.83-07 for OS and PS, respectively) (**Figure 5A**). We further performed univariate COX regression analyses by including patient age, gender, stage, and grade together with the CIN25 clustering system. Advanced Stages, higher grades and CIN25-C2 were all associated with shorter OS and PFS (**Figures 5B, C**). Multivariate COX regression analyses showed that all three of them were independent prognostic factors for shorter OS and PFS (**Figures 5B, C**).

The E-MTAB-1980 ccRCC cohort as the validation set were further analyzed in the same manner. There was no PFS information available, and we only evaluated OS. Kaplan–Meier survival analysis showed that CIN25-C2 was associated with significantly shorter OS (*P* = 0.0003) (**Figure 5D**), and the CIN25 subtype and stages were independent OS predictors, as assessed using univariate (**Figure 5E**) and multivariate COX regression analyses (**Figure 5F**).

The data above consistently show that CIN25-C2 subtype and advanced stages are independent prognostic variables for OS and/or PFS in both TCGA and E-MTAB-1980 cohorts. We thus established a prognostic nomogram composed of CIN25 subtypes and stages. For the TCGA cohort, the nomograms exhibited a highly accurate estimation of OS and PFS possibilities at 1-, 3- and 5-years (**Figures S2A, B**). Similar results were obtained for OS prediction in the E-MTAB-1980 cohort (**Figure S2C**). To further evaluate the sensitivity and specificity of their prediction, we did time-dependent ROC analyses. In the TCGA cohort, Area under ROC curves (AUCs) for 1-, 3- and 5-year OS were 0.799, 0.767 and 0.740, respectively, while the AUCs for 1-, 3- and 5-year PFS were 0.825, 0.797 and 0.798, respectively (**Figures S2D, E**). For 1-, 3- and 5-year OS in the E-MTAB-1980 cohort, AUCs were 0.886, 0.871 and 0.838, respectively (**Figure S2F**). Separate analyses of these two variables showed that AUCs were largely between 0.6 and 0.7, between 0.7 and 0.8 for all CIN25- and stage-based predictions of 1-, 3- and 5-year survival (**Figures S2G, H**). AUCs obtained from stage-prediction were bigger in all the estimations.

### The CIN25 cluster as a predictor for patient response to Sunitinib treatment

**Figure 6:**
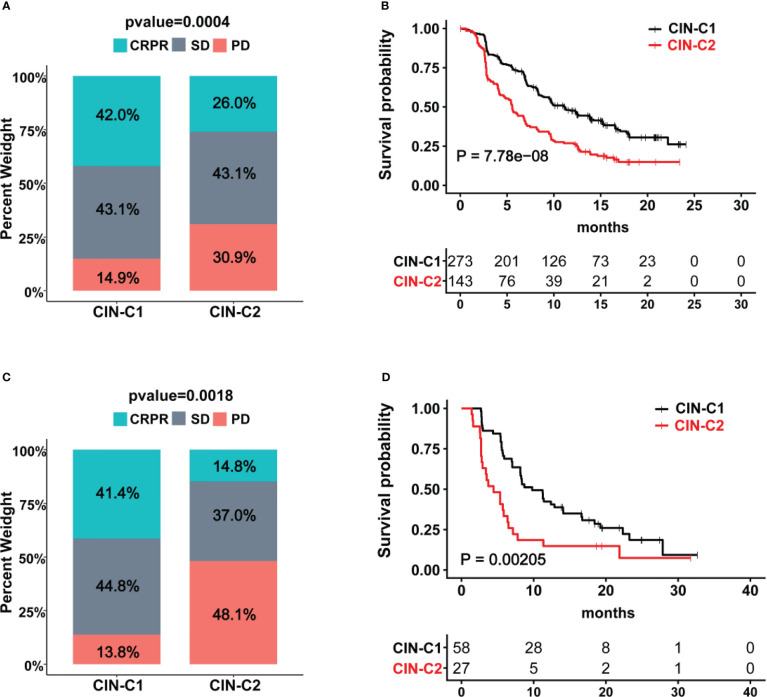
The CIN25 subtypes for prediction of Sunitinib response in ccRCCs. **(A, B)** IMmotion151 cohort of ccRCC patients treated with Sunitinib. Poor response to Sunitinib and shorter patient PFS in the CIN25-C2 group. **(C, D)** IMmotion150 cohort of ccRCC patients treated with Sunitinib. Poor response to Sunitinib and shorter patient PFS in the CIN25-C2 group.

Sunitinib has long been applied for advanced ccRCC treatment as the first line drug (14), however, reliable biomarkers to predict its efficacy or patient response are few (6, 8). We thus determined whether the CIN25 cluster classification could help distinguish responders from non-responders in patients treated with Sunitinib. Toward this end, the IMmotion151 cohort of 416 ccRCC patients treated with Sunitinib was first analyzed as the discovery set (31, 32). Patient response to Sunitinib was categorized into complete remission (CR), partial remission (PR), stable disease (SD) and progressive disease (PD), respectively. A total of 416 patients were classified into CIN25-C1 (273 patients) and C2 (143 patients) groups. The CR and PR (CRPR) rate was 42% and 26% in CIN25-C1 and C2 groups, respectively (*P* = 0.0004) (**Figure 6A**). The disease progression during the Sunitinib treatment occurred in 14.9% and 30.9% for CIN25-C1 and C2 patients, respectively. The median PFS for C1 and C2 patients was 5.6 and 11.2 months, respectively (*P* = 7.78E-08; HR, 1.90 (95% CI: 1.45 – 2.47) (**Figure 6B**). We then analyzed the IMmotion150 cohort (29, 30) to validate the findings obtained from IMmotion151 cohort. In a total of 85 available patients, CIN25-C1 and C2 were 58 and 27, respectively. The total CRPR rate was 41.4% and 14.8% in CIN25-C1 and C2 groups, respectively (*P* = 0.002) (**Figure 6C**). Almost the half of CIN25-C2 patients (48.1%) underwent progression during the treatment, while only 13.8% of CIN25-C1 patients did so (**Figure 6C**). Higher CRPR rates in CIN25-C1 group led to longer PFS, and the median PFS for C1 and C2 patients was 4.4 and 9.8 months, respectively (*P* = 0.002; HR, 2.13 (95% CI: 1.18 – 3.84) (**Figure 6D**).

### Signaling pathways enriched in CIN25-C2 tumors and phenotypic association

**Figure 7:**
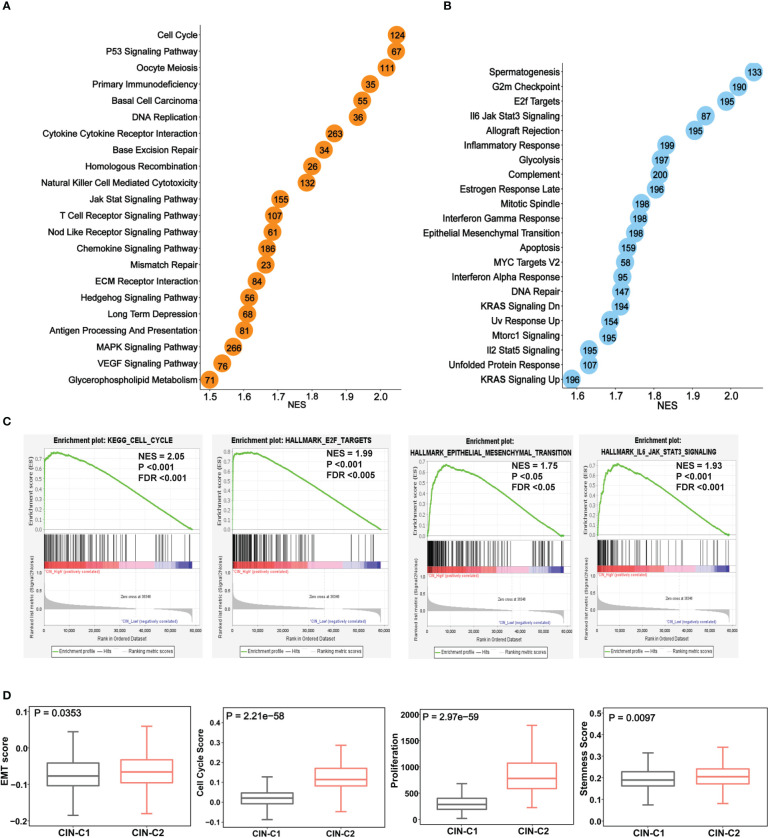
The enriched oncogenic pathways and aggressive phenotypes in the CIN25-C2 subtype of ccRCC tumors. The TCGA cohort analysis. **(A, B)** Enrichments of overrepresented KEGG **(A)** and hallmark **(B)** pathways in CIN25-C2 tumors. **(C)** Representative enriched pathways in CIN25-C2 tumors: Cell cycle, E2F targets, EMT and IL6-JAK-STAT3. **(D)** Higher EMT, proliferation and stemness scores in CIN-C2 tumors.

We next performed the GSEA analysis to probe differences in signaling pathways between two tumor groups. **Figures 7A, B** showed significantly enriched KEGG and hallmark pathways in CIN25-C2 tumors, and almost all of them are oncogenic and play key parts in ccRCC development and progression, such as G2/M checkpoint, E2F and MYC targets, IL6-JAK-STAT3, glycolysis, EMT and others (**Figure 7C**). Consistent with these enriched pathways, CIN25-C2 tumors had robustly strong proliferation activity compared to CIN25-C1 tumors, as assessed using proliferation marker Ki-67 and cell cycle score, and stemness score (**Figure 7D**); furthermore, an established EMT 16 gene signature (36) was further used to evaluate EMT between CIN25-C1 and C2 tumors and significantly increased EMT scores were observed in the CIN25-C2 group (**Figure 7D**) (*P* = 0.035).

### Increased EZH2 expression and diminished angiogenesis in CIN25-C2 tumors

**Figure 8:**
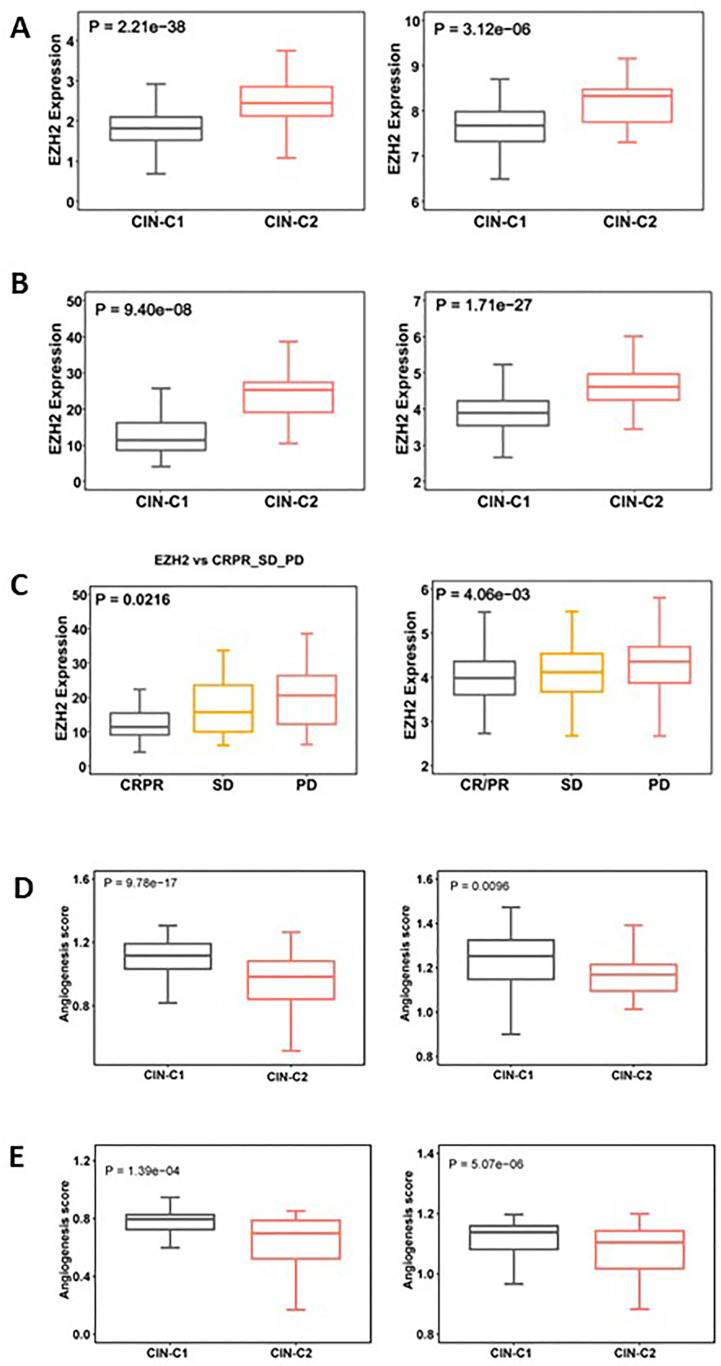
Differences in EZH2 expression and angiogenesis between CIN25-C1 and C2 subtypes of ccRCC tumors. **(A, B)** CIN25-C2 tumors expressed significantly higher levels of EZH2 mRNA. **(A)** TCGA (left) and E-MTAB1980 (right) ccRCC cohorts. **(B)** IMmotion150 (left) and 151 (right) cohorts. **(C)** Differences in EZH2 expression in tumors from CRPR, SD and PD patients (left and right: IMmotion150 and 151 cohorts, respectively). **(D)** Lower angiogenesis scores in CIN25-C2 tumors (left and right: TCGA and E-MTAB1980 ccRCC cohorts, respectively). **(E)** Lower angiogenesis scores in CIN25-C2 tumors (left and right: IMmotion150 and 151 cohorts, respectively).

EZH2, a histone methyltransferase catalyzing H3K27 trimethylation (H3K27me3), has been shown to promote stemness, EMT and Sunitinib resistance in ccRCC and other tumors (42–44). Given the results above, we set out to determine whether EZH2 expression differed between CIN25-C1 and C2 tumors. The analysis of both TCGA and E-MTAB1980 ccRCC cohorts showed robustly higher EZH mRNA levels in CIN25-C2 than in C1 tumors (CIN25-C1 vs C2: *P* = 2.21E-38 and 3.12E-06, respectively) (**Figure 8A**). In the Sunitinib-treated IMmotion150 and IMmotion151 cohorts, similar results were obtained (CIN25-C1 vs C2: *P* = 9.40E-08 and 1.71E-27 for IMmotion150 and 151, respectively) (**Figure 8B**). We further compared differences in EZH2 expression between responders and non-responders to Sunitinib. As expected, tumors from resistant patients expressed significantly higher levels of EZH2 than did those from responders (*P* = 0.021 and 0.004, respectively) (**Figure 8C**).

In addition to higher EZH2 expression, poor angiogenesis is also a well characterized predictor for Sunitinib resistance (34, 35), and we thus analyzed the angiogenesis score in CIN25 subtypes of ccRCC tumors. As shown in **Figure 8D**, a significantly lower angiogenesis score was observed in the CIN-C2 tumors from the IMmotion151 (CIN25-C1 vs C2: *P* = 9.78E-17) and IMmotion150 (*P* = 0.0096) cohorts of ccRCC patients treated with Sunitinib. The TCGA and E-MTAB1980 ccRCC analyses showed similar results, which validated the observations above (**Figure 8E**).

## Discussion

CIN is an important cancer hallmark (23–25). Because of the difficulty in directly assessing a CIN phenotype, a CIN25 signature has been developed, and the CIN25 expression-based score system roughly reflected levels of CIN in several cancer types (22). By analyzing primary ccRCC tumors and TCGA ccRCC cohort, we observed that expression of genes included in the CIN25 signature was robustly upregulated but significantly heterogenous. Based on CIN25 scores calculated from their mRNA levels, we categorized ccRCC patients into two clusters: CIN25-C1 (CIN25-low) and CIN25-C2 (CIN25-high), respectively. Our findings demonstrate that the CIN25 signature is present in ccRCC and this cluster system is useful in predicting patient outcomes and therapeutic response to TKR inhibitors.

CIN has been shown as a key driver of chromosomal alterations in human malignancies and primarily characterized by aneuploidy or SCNAs (23–25). Consistent with this, we observed that CIN25-C2 ccRCC tumors exhibited robustly increased aneuploidy. CIN-triggered aneuploidy creates intratumour genetic heterogeneity, thereby promoting phenotypic adaptation during cancer evolution and progression. On the other hand, aneuploidy or SCNAs further accelerate CIN rates. Thus, CIN and aneuploidy affect each other, establishing positive feedback.

CIN underpins much of the intratumoural heterogeneity observed in cancers and drives phenotypic adaptation during tumor evolution (23–25). It has been shown that the CIN phenotype is associated with resistance to chemo- and radio-therapies, however, it remains to be defined whether it has impacts on targeted therapeutic drugs. Sunitinib, a TKR inhibitor, has been applied as the first-line drug for advanced ccRCC treatment (12–14). Clinical studies showed that the intrinsic resistance to Sunitinib occurred in approximately 1/3 of patients, while many of them initially responded to Sunitinib but the treatment failure developed eventually (6, 12, 15). Several molecules and signaling pathways have been implicated in Sunitinib irresponsiveness, however, the development of reliable biomarkers that distinguish Sunitinib responders from non-responders remains challenging. Our present findings suggest that the CIN25 signature serves as a useful stratifier to predict the therapeutic efficacy of Sunitinib and PFS in ccRCC patients. EZH2 upregulation and poor angiogenesis are likely the mechanism underlying lower efficacy observed in CIN25-C2 patients. Further studies are required to elucidate how CIN25 signature affects EZH2 expression and angiogenesis.

A link between telomere dysfunction and CIN has been well characterized in human malignancies and animal carcinogenesis models (39). Telomeric DNA repeats, when sufficient long, together with their binding-factors or sheltering proteins, form protective structures at the ends of linear chromosomes that prevent CIN (39, 45). Telomeric DNA is synthesized by telomerase, an RNA-dependent DNA polymerase activated in most human malignancies for telomere length maintenance (40). However, telomerase activation usually occurs at the late stage during a stepwise malignant transformation (45). Therefore, telomeres already become shortened in precursor lesions, which leads to telomere dysfunction as a driving event for CIN in early carcinogenesis (39, 45, 46). Shorter or dysfunctional telomere-bearing chromosomes are prone to fusion, thereby triggering the dicentric chromosome formation that missegregate or break in mitosis during anaphase (39). The resultant chromosomal breaks are fusogenic, through which a cycle of chromosome fusion and breakage is propagated. In the present study, we observed significantly shorter telomeres in ccRCC tumors than in their matched renal tissues. There were no differences in telomere length between CIN25-C1 and C2 tumors, but TERT expression and telomerase activity was noticeably higher in CIN25-C2 tumors. Likely, increased telomerase activity attenuates or compensates for telomere attrition in CIN25-C2 tumors.

CIN is one subtype in the genomic instability category that encompasses a variety of DNA alterations, including single nucleotide to whole chromosome changes (41). Interestingly, we observed that CIN25-C2 tumors also had increased genomic alterations reflecting all other aspects of genomic instability. In addition, HRD has been implicated in genomic instability including CIN, and consistently, HRD scores were significantly higher in CIN25-C2 tumors. Thus, the CIN25 clustering system help measure not only the CIN phenotype, but also the whole genomic instability level. From this point of a view, assessment of CIN25 signature may have broader implications both biologically and clinically. For instance, HRD occurs frequently in breast and ovarian cancer, and those patients are in general sensitive to PARP inhibitors. Conceivably, the CIN25 assessment may also be useful to stratify patients who respond to PARP inhibitor treatment. A PCR method is sufficient to quantify expression levels of 25 CIN genes, which is cost- and time-friendly, and easily applied for clinical routine.

In conclusion, the CIN25 clustering model can categorize ccRCC tumors into CIN25-C1 and C2 subtypes, and this classification hold great promises in predicting patient survival and response to Sunitinib. CIN25-C2 tumors are characterized by active proliferation, stemness and EMT phenotypes. EZH2 overexpression and poor angiogenesis may drive all these aggressive phenotypes, shorter survival and drug resistance. Importantly, the CIN25 clustering model not only represents a CIN phenotype, but also is strongly associated with other genomic instability-related alterations. Thus, the assessment of CIN25 reflects levels of CIN and whole genomic instability. Moreover, a PCR quantification is enough for the CIN25-based tumor classification, which is suitable for clinical routine application. Taken together, the present findings will contribute to improved personalized management of ccRCCs.

## Data availability statement

The datasets presented in this study can be found in online repositories. The names of the repository/repositories and accession number(s) can be found in the article/**Supplementary Material**.

## Ethics statement

The studies involving human participants were reviewed and approved by Institutional review board of Qilu Hospital of Shandong University. The patients/participants provided their written informed consent to participate in this study.

## Author contributions

All authors participated in the conception and design of the study. CW, HY, JW, XQ, LL and WG performed bioinformatic analysis. XQ and ZF analyzed patient data and RNA sequencing. CW, XQ, HY, ZF, YF and DX participated in the data process, analysis and interpretation. CW, XQ, HY, YF and DX conceived and drafted the manuscript. HY, YF and DX revised the manuscript. All authors contributed to the article and approved the submitted version.
