# Supplementary material for: The chromosomal instability 25 gene signature is identified in clear cell renal cell carcinoma and serves as a predictor for survival and Sunitinib response
Source: Front Oncol. 2023 May 1;13:1133902. doi: 10.3389/fonc.2023.1133902 (PMC10183591; doi:10.3389/fonc.2023.1133902)
Supplement: Supplementary file 1 [file DataSheet_1.pdf]

The chromosomal instability 25 gene signature is identified in clear cell renal cell carcinoma and serves as a predictor for survival and sunitinib response

Chang Wang[1,2], Xin Qin[2], Wei Guo[2], Jing Wang[3], Li Liu[4], Zhiqing Fang[2], Huiyang Yuan[2], Yidong Fan[2], Dawei Xu[5]

SUPPLEMENTARY MATERIAL

Include Table S1 – S6 and Figure S1, S2 and S2.

***Table S1-1**. Characteristic of Qilu Hospital Cohort of 10 ccRCC patients for RNA seq.*

Qilu Hospital Cohort	***N=10***
Age, mean (SEM[a])	52.7 (10.3)
Sex, n (%)	
Female	3 (30.0%)
Male	7 (70.0%)
AJCC Stage[b], n (%)	
I	5 (50.0%)
II	1 (10.0%)
III	4 (40.0%)
T, n (%)	
T1	5 (50.0%)
T2	1 (10.0%)
T3	4 (40.0%)
Lymph node metastasis, n (%)	
N0	9 (90.0%)
N1	1 (10.0%)
Grade, n (%)	
G1/G2	5 (50.0%)
G3/G4	5 (50.0%)
Tumor max diameter, n (%)	
<4	2 (20.0%)
4-7	4 (20.0%)
7-10	2 (40.0%)
>10	2 (20.0%)

[a]SEM, standard error of mean.

[b]Tumor AJCC stages according to the American Joint Committee on Cancer (AJCC) 7th edition.

Table S1-2. Characteristics of 9 ccRCC patients with matched tumor and non-tumorous tissues for qPCR analysis.

Variable	N
Age, mean (SEM[a])	55.2 (3.4)
Sex, n (%)	
Male	6 (66.7)
Female	3 (33.3)
Grade, n (%)	
Low	4 (44.4)
High	5 (55.6)
AJCC Stage[b], n (%)	
I	4 (44.4)
II	1 (11.1)
III	4 (44.4)
T, n (%)	
$<$ T2	4 (44.4)
$\geqslant$ T2	5 (55.6)
Lymph node metastasis, n (%)	
Positive	1 (11.1)
Negative	8 (88.9)

[a]SEM, standard error of mean.

[b]Tumor AJCC stages according to the American Joint Committee on Ca

Table S2. qPCR primer sequences used in the present study

H-FOXM1-194F	GGCCATCCCCAACAATGCTA
H-FOXM1-194R	AGGTCTCCAGGGTCACTTCT
H-CDC2-112F	AGTCAGTCTTCAGGATGTGCT
H-CDC2-112R	CATGTACTGACCAGGAGGGAT
H-PRC1-152F	TGTGGCAGAACAATGGGAGAT
H-PRC1-152R	GTGTATTGGGAGCCAGTCCTC
H-TPX2-236F	CCAGAGAAAGCCAAGGGTAGA
H-TPX2-236R	TCTGTGCGGAAGTGGAAGTC
H-MCM2-116F	ATCTACGCCAAGGAGAGGGT
H-MCM2-116R	GTAATGGGGATGCTGCCTGT
H-MCM7-171F	GAACACAAGGATTGCCCAGC
H-MCM7-171R	CCAGCCCCAGACTCATCATC
H-KIF20A-165F	AAGGGCAGAACTGGCTCATC
H-KIF20A-165R	GCAAGGGCTTCAGATCAGGT
H-TOP2A-168F	TCAGGCGTTTGATGGATGGA
H-TOP2A-168R	TCCATGTTCTGACGGGAAGC
H-TTK-130F	TGCCCATTTGGAAGAGTCCC
H-TTK-130R	GCACAACCAAATCTCGGCAT
H-ESPL1-105F	TGATTTGGCTGACCTGACCC
H-ESPL1-105R	ATGTGGTCCGTCAGCTCTTG
H-NCAPD2-177F	GATGCGTAATGCTGTGCTGG
H-NCAPD2-177R	ATTCGGGTGAAGAGCTGCAA
H-MELK-92F	AGGTGGACCCAAAGAAACGG
H-MELK-92R	GCTTTGCCACTCAACAGGAT
H-UBE2C-134F	CCTTGAACACACATGCTGCC
H-UBE2C-134R	AAGACGACACAAGGACAGGC
H-FEN1-166F	CCAACAAGTACCCTGTGCCA
H-FEN1-166R	CGCTCCTCAGAGAACTGCTT
H-RFC4-190F	TTACGGACCACCTGGAACTG
H-RFC4-190R	CTTCCCATCTGAGCGACTTC
H-CDC45-133F	GATGTTTGAGCTGGCTTGGAT
H-CDC45-133R	CAGGACACCAACATCAGTCAC
H-RAD51AP1-92F	GATGTTGGTGGTGTTCAAGGGA
H-RAD51AP1-92R	CTATCACCATCACTGCCTTCCA
H-H2AZ1-211F	TCGAAATGGCTGGCGGTAAG
H-H2AZ1-211R	AGTTCAAGTACCTCTGCGGTG
H-PCNA-71F	GCTCCATCCTCAAGAAGGTGT
H-PCNA-71R	GGAGCTAATATCCCAGCAGGC
H-RNASEH2A-F	TCAGGCTACCCCAATGATCC
H-RNASEH2A-R	CCTCCTGATTCTCGGATGCT
H-TGIF2-206F	GATCTAGGTGAGGACGAAGGC

H-TGIF2-206R	CGGGCATTGATGAACCAGTTAC
H-CCT5-151F	CAAGATCAGCGATAGCGTCCT
H-CCT5-151R	CGGCATTCACAGCAATCTCAG
H-TRIP13-115F	TGGCACTGGAAAAACATCCCT
H-TRIP13-115R	AGAAAAGAGGCTGTGGCTGTT
H-MAD2L1-222F	TCTCATTCGGCATCAACAGCA
H-MAD2L1-222R	CCAGGACCTCACCACTTTCAA
H-CCNB2-172F	CGACGGTGTCCAGTGATTTG
H-CCNB2-172R	TTGGTGGGTTGAACTGGAACT
homoβ-actin-250 F	CATGTACGTTGCTATCCAGGC
homoβ-actin-250 R	CTCCTTAATGTCACGCACGAT

Table S3*. Clinic-Pathological characteristic of TCGA ccRCC Cohort*

	**CIN-C1** *N=350*	**CIN-C2** *N=180*	**P**
Age, mean (SEM[a])	61.4 (12.2)	59.3 (11.8)	0.055
Sex, n (%)			0.063
Male	217 (62.0%)	127 (70.6%)	
Female	133 (38.0%)	53 (29.4%)	
AJCC Stage[b], n (%)			<0.001
I	196 (56.0%)	70 (39.5%)	
II	43 (12.3%)	14 (7.91%)	
III	74 (21.1%)	49 (27.7%)	
IV	37 (10.6%)	44 (24.9%)	
Grade, n (%)			<0.001
G1	12 (3.49%)	2 (1.12%)	
G2	167 (48.5%)	61 (34.3%)	
G3	133 (38.7%)	73 (41.0%)	
G4	32 (9.30%)	42 (23.6%)	

[a]SEM, standard error of mean.

[b]Tumor AJCC stages according to the American Joint Committee on Cancer (AJCC) 7th edition.

***Table S4**. Clinic-Pathological characteristic of E-MTAB-1980 ccRCC Cohort*

	**CIN-C1**<br>***N=64***	**CIN-C2**<br>***N=37***	**P**
Age, mean (SEM[a])	62.3 (11.6)	65.5 (11.1)	0.172
Sex, n (%)			0.010
Male	43 (67.2%)	34 (91.9%)	
Female	21 (32.8%)	3 (8.11%)	
AJCC Stage[b], n (%)			0.125
I	46 (71.9%)	20 (54.1%)	
II	7 (10.9%)	3 (8.11%)	
III	5 (7.81%)	8 (21.6%)	
IV	6 (9.38%)	6 (16.2%)	
Grade, n (%)			0.007
G1	10 (15.9%)	3 (8.33%)	
G2	43 (68.3%)	16 (44.4%)	
G3	9 (14.3%)	13 (36.1%)	
G4	1 (1.59%)	4 (11.1%)	

[a]SEM, standard error of mean.

[b]Tumor AJCC stages according to the American Joint Committee on Cancer (AJCC) 7th edition.

***Table S5**. Characteristic of IMmotion151 Cohort of Sunitinib treatment*

	**CIN-C1** ***N=273***	**CIN-C2** ***N=143***	**P**
Age, mean (SEM[a])	60.0 (10.1)	59.3 (9.63)	0.470
Sex, n (%)			0.223
Female	62 (22.7%)	41 (28.7%)	
Male	211 (77.3%)	102 (71.3%)	
Histology			<0.001
ccRCC_nonSarc	245 (89.7%)	101 (71.1%)	
ccRCC_Sarc	26 (9.52%)	30 (21.1%)	
nonccRCC_Sarc	2 (0.73%)	11 (7.75%)	
Response:			<0.001
CR/PR	107 (42.0%)	32 (26.0%)	
PD	38 (14.9%)	38 (30.9%)	
SD	110 (43.1%)	53 (43.1%)	

[a]SEM, standard error of mean.

Table S6*. Characteristic of IMmotion151 Cohort of Sunitinib treatment*

	**CIN-C1**<br>***N=58***	**CIN-C2**<br>***N=27***	**P**
Age, mean (SEM[a])	60.1 (9.88)	60.9 (12.0)	0.763
Sex, n (%)			1.000
Female	10 (17.2%)	5 (18.5%)	
Male	48 (82.8%)	22 (81.5%)	
Tumor Mutation Burden	26.0 (106)	11.4 (10.8)	0.385
Response:			0.001
CR/PR	24 (41.4%)	4 (14.8%)	
PD	8 (13.8%)	13 (48.1%)	
SD	26 (44.8%)	10 (37.0%)	

[a]SEM, standard error of mean.

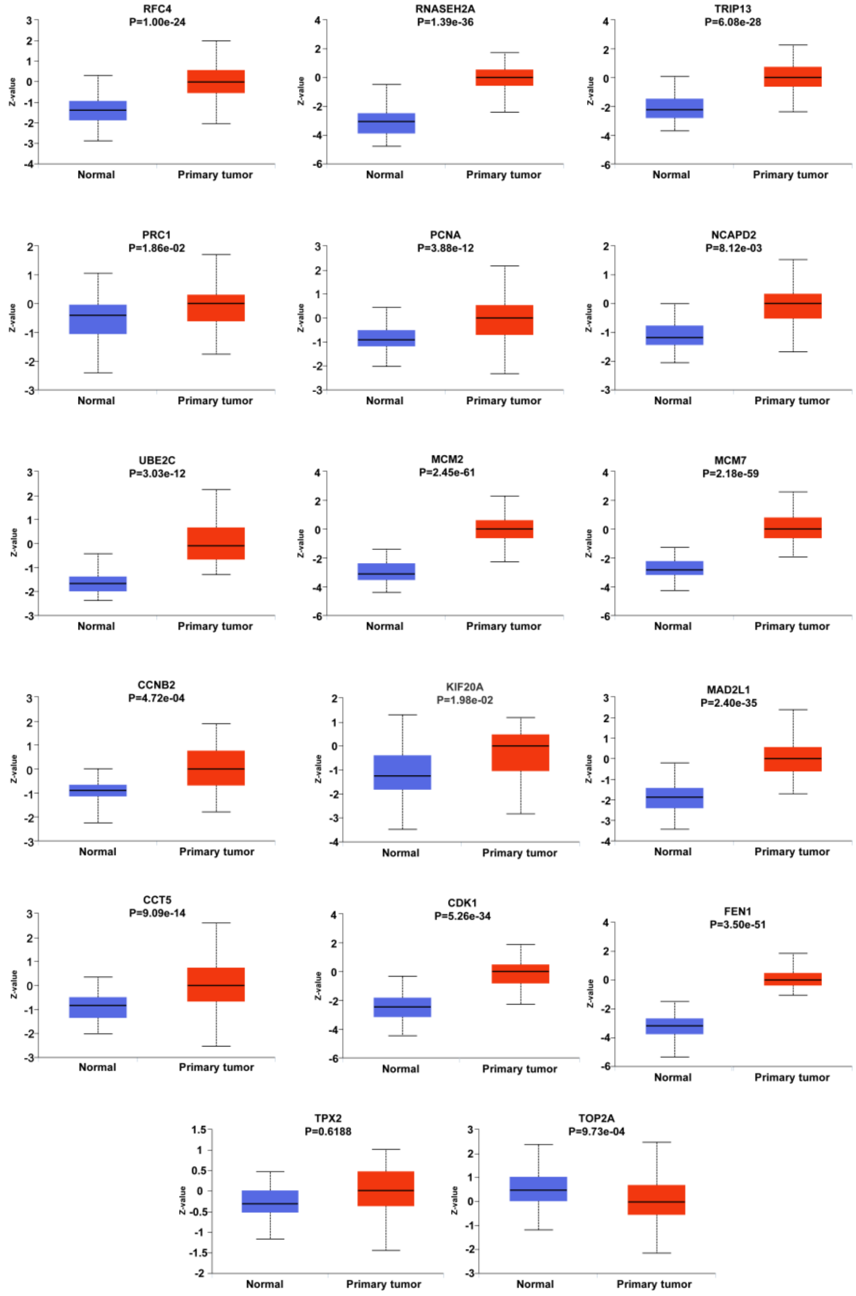

Figure S1. Expression of CIN25 genes at protein levels in ccRCC tumors and renal nontumorous tissues in the TCGA cohort. The data were obtained from Clinical Proteomic Tumor Analysis Consortium (http://ualcan.path.uab.edu/index.html), and 17 of 25 genes were available. Higher protein levels in tumors were observed in 16/17 genes.

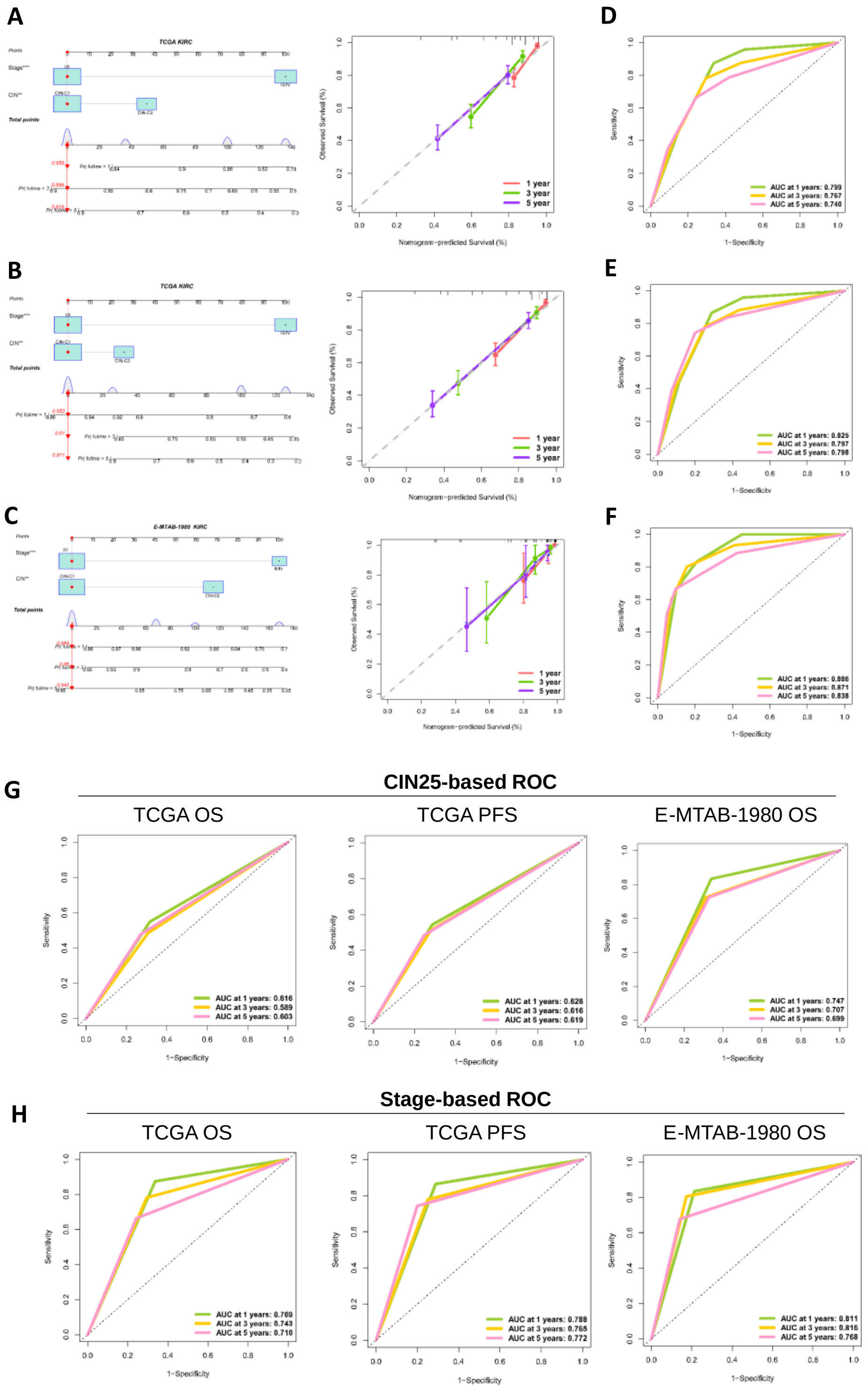

Figure S2. CIN25 subtype- and stage-based nomograms and time-dependent Receiver Operator Characteristic (ROC) to predict overall and progression-free survival (OS and PFS) in TCGA and E-MTAB-1980 cohorts of ccRCC patients. (A) Nomogram prediction of 1-, 3- and 5-year OS in TCGA cohort. (B) Nomogram prediction of 1-, 3- and 5-year PFS in TCGA cohort. (C) Nomogram prediction of 1-, 3- and 5-year OS in E-MTAB-1980 cohort. (D) ROC of CIN25- and stage-based 1-, 3- and 5-year OS prediction in TCGA cohort. (E) ROC of CIN25- and stage-based 1-, 3- and 5-year PFS prediction in TCGA cohort. (F) ROC of CIN25- and stage-based 1-, 3- and 5-year OS prediction in E-MTAB-1980 cohort. (G) ROC of CIN25-based 1-, 3- and 5-year survival prediction in both cohorts. (H) ROC of stage-based 1-, 3- and 5-year survival prediction in both cohorts.

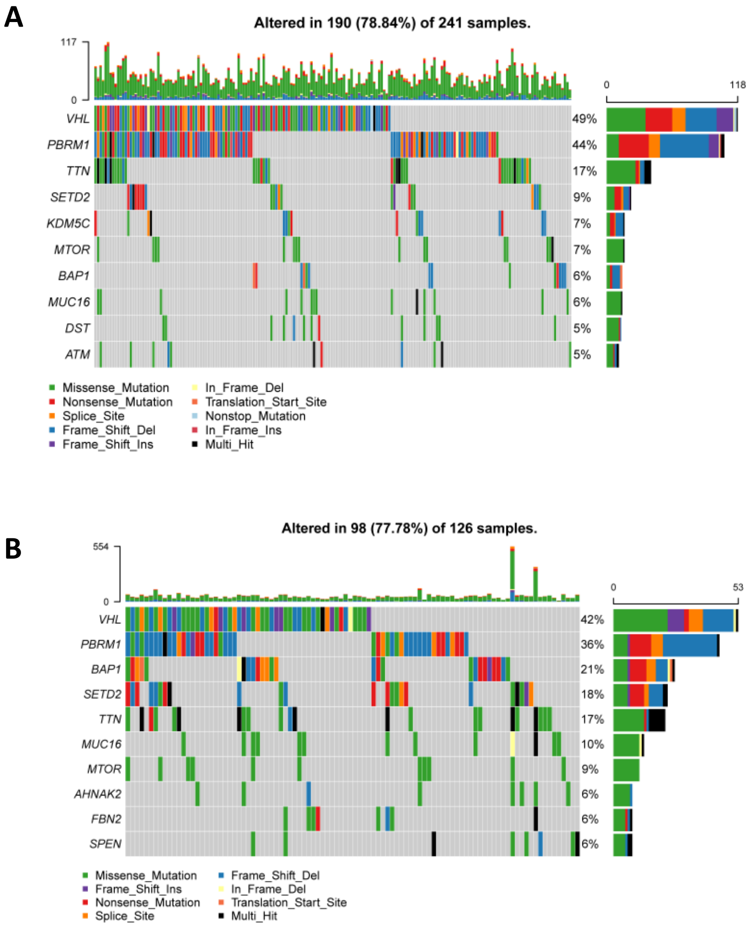

Figure S3. The top 10 mutated genes in CIN25-C1 and C2 tumors from the TCGA ccRCC cohort. (A) CIN25-C1. (B) CIN25-C2.
